# Magnetic Field Meter Based on CMR-B-Scalar Sensor for Measurement of Microsecond Duration Magnetic Field Pulses

**DOI:** 10.3390/s25061640

**Published:** 2025-03-07

**Authors:** Pavel Piatrou, Voitech Stankevic, Nerija Zurauskiene, Skirmantas Kersulis, Mindaugas Viliunas, Algirdas Baskys, Martynas Sapurov, Vytautas Bleizgys, Darius Antonovic, Valentina Plausinaitiene, Martynas Skapas, Vilius Vertelis, Borisas Levitas

**Affiliations:** 1 Department of Functional Materials and Electronics, Center for Physical Sciences and Technology, Sauletekio ave. 3, LT-10257 Vilnius, Lithuania; pavel.piatrou@ftmc.lt (P.P.); nerija.zurauskiene@ftmc.lt (N.Z.); skirmantas.kersulis@ftmc.lt (S.K.); algirdas.baskys@ftmc.lt (A.B.); martynas.sapurov@ftmc.lt (M.S.); vytautas.bleizgys@ftmc.lt (V.B.); darius.antonovic@ftmc.lt (D.A.); valentina.plausinaitiene@ftmc.lt (V.P.); martynas.skapas@ftmc.lt (M.S.); vilius.vertelis@ftmc.lt (V.V.); 2 Faculty of Electronics, Vilnius Gediminas Technical University, Plytines Str. 25, LT-10105 Vilnius, Lithuania; 3 Faculty of Physics, Vilnius University, Sauletekio al. 9, LT-10222 Vilnius, Lithuania; mindaugas.viliunas@ff.vu.lt; 4 Geozondas Ltd., Sevcenkos Str. 16 B, LT-03111 Vilnius, Lithuania; levitas@geozondas.com

**Keywords:** magnetic field sensors, colossal magnetoresistance, magnetic field measurement system, parasitic electromotive force, pulsed magnetic field, magnetic field dynamics, magnetic pulse welding

## Abstract

This study presents a system for precisely measuring pulsed magnetic fields with high amplitude and microsecond duration with minimal interference. The system comprises a probe with an advanced magnetic field sensor and a measurement unit for signal conversion, analysis, and digitization. The sensor uses a thin nanostructured manganite La-Sr-Mn-O film exhibiting colossal magnetoresistance, which enables precise magnetic field measurement independent of its orientation. Films with different compositions were optimized and tested in pulsed magnetic fields. The measurement unit includes a pulsed voltage generator, an ADC, a microcontroller, and an amplifier unit. Two versions of the measurement unit were developed: one with a separate amplifier unit configured for the sensor positioned more than 1 m away from the measurement unit, and the other with an integrated amplifier for the sensor positioned at a distance of less than 0.5 m. A bipolar pulsed voltage supplying the sensor minimized the parasitic effects of the electromotive force induced in the probe circuit. The data were transmitted via a fiber optic cable to a PC equipped with a special software for processing and recording. Tests with 20–30 μs pulses up to 15 T confirmed the effectiveness of the system for measuring high pulsed magnetic fields.

## 1. Introduction

Pulsed magnetic fields play an important role in various scientific and industrial applications, such as plasma research, nondestructive pulsed magnets, magnetic flux compression, magnetic pulse welding, electromagnetic launchers, and particle accelerators [1]. The measurement of these fields, especially if they are short in duration and large magnitudes, faces unique challenges. The choice of measurement methods depends on factors such as field strength, temporal variation, homogeneity, and required accuracy.

In general, pulsed magnetic fields can be measured using the method of time derivative of the magnetic field (dB/dt) or by direct measurement of the magnitude of the magnetic field [2,3,4,5,6,7]. Sensors based on the time derivative, such as B-dot sensors (induction coils), are widely used as they are capable of generating high output voltages when exposed to fields with large dB/dt values. This is a common characteristic of short pulsed magnetic fields. These sensors have already been successfully used in plasma experiments, railguns, and coilguns [8,9,10]. However, the signal from these sensors must be integrated to reconstruct the actual waveform of the magnetic field, leading to potential errors caused by noise accumulation during the integration process. Direct techniques for measuring the magnetic field, such as Hall effect sensors and magnetoresistive sensors based on anisotropic magnetoresistance (AMR), giant magnetoresistance (GMR), and tunneling magnetoresistance (TMR) effects, bypass the need for integration but have their limitations [11,12]. They are susceptible to noise induced by large dB/dt rates, and saturation effects limit their use to strong magnetic fields [13]. In addition, most of these sensors are sensitive to the direction of the magnetic field, requiring precise alignment or multiple orthogonally arranged sensors to measure the vector components of the field [14,15,16,17]. While three-dimensional sensor arrays can solve the problem of directionality, they present additional challenges, such as bulky setups, increased wiring complexity, and induced voltage errors in short-pulse measurements.

Recent advances in sensor technology have opened up new possibilities to overcome the limitations of conventional sensors. One notable innovation is the development of CMR-B scalar sensors based on the effect of colossal magnetoresistance (CMR) [18] in polycrystalline manganite films (La-Sr-Mn-O) [19,20]. These sensors offer several advantages for the measurement of pulsed magnetic fields. They are direction-independent, compact and can operate in small volumes. In contrast to direction-dependent sensors, CMR-B scalar sensors do not need to be precisely aligned with the magnetic field, simplifying their installation in experimental setups. They have been successfully used to measure magnetic field distributions in railguns and coilguns, as well as during magnetic pulse welding and pulsed field magnets with amplitudes in the megagauss range [21,22,23].

A critical problem in measuring short-pulse magnetic fields is the electromotive force (EMF) induced in the transmission lines or sensor wires [13]. This EMF is caused by rapid changes in the magnetic field, as described by Faraday’s law of induction, which distorts the sensor signal and affects measurement accuracy. The effect is particularly pronounced for short pulses where the magnetic field’s rate of change is extremely high and generates significant unwanted voltage signals in the connecting cables. There are various techniques to minimize or eliminate this interference. One of these is the use of twisted pair cables [24]. It helps to reduce EMF interference by ensuring that the induced voltages in neighboring cables largely cancel each other out. This is a simple and effective method of reducing the noise caused by external magnetic fields. However, if the EMF value is many times greater than the signal of the measured magnetic field, this method is ineffective. An additional loop can also be inserted in the signal transmission line to compensate for the induced EMF [19]. By designing the compensation loop to generate an opposite voltage, the net EMF in the signal line can be minimized. However, the problematic positioning of this loop relative to the sensor does not lead to effective results. Other methods, such as changing the magnetic field’s direction or modulating the AC supply to the sensor, also have significant limitations in their application for EMF compensation [25].

Recently, a method for measuring short magnetic fields with durations in the order of 20–100 μs was proposed in [26]. In the proposed method, the sensor was supplied with a bipolar voltage in the form of square-wave pulses. Measuring the positive and negative voltages across the sensor and subsequently processing this signal according to Equation (1) made it possible to eliminate this electromotive force induced in the wires connecting the sensor to the measuring unit.

*V*(*B*) = [(+*V*(*B*) + EMF) − (−*V*(*B*) + EMF)]/2. (1)

In this equation, +*V* and −*V* are the voltages of two adjacent pulses. When the sensor is supplied by positive and negative voltages, respectively [26], *V*(*B*) is the useful response signal of the sensor, which is directly related to the sensor’s resistance change due to a change of magnetic field *B*.

Nevertheless, there are some problems with such a methodology. One of them is that the cable connecting the sensor to the measurement module has a specific impedance in which the capacitive component plays a major role. In this case, the voltage across the sensor is not rectangular-shaped but has an exponentially increasing and decreasing character. The higher the frequency of the pulsed supply voltage, the higher the resistance of the sensor itself, and the higher the capacitance of the cable, the longer the time it takes for the voltage across the sensor to achieve the supply voltage. At the same time, the frequency of the sensor’s bipolar power supply must be at least 20–50 times higher than the frequency of the measured field to precisely measure the pulsed magnetic field. In addition, the capacitance of the cable, which depends on its length and configuration, must be reduced to a minimum.

One of the basic parameters that can reduce the distortion of the sensor’s supply voltage is the electrical resistance of the sensor itself. As mentioned above, CMR-based sensors are perfect candidates for use in pulsed magnetic field measurements. In addition to their parameters, such as independence of the direction of the magnetic field, their advantage lies in the possibility to adopt their parameters for the needs of measurement. It was shown that for nanostructured polycrystalline films of manganite La_1−x_Sr_x_Mn_y_O_3_, the change of the doping level of Sr and Mn and the tuning of these films’ growth conditions can be used to control the resistivity, magnetoresistance, and other characteristics of these films [26,27,28]. For example, the excess manganese content in manganite films could result in reduced resistivity and increased magnetoresistance values at higher than room temperatures, making these materials suitable for measurements over a wide temperature range.

In this paper, we present the design and test results of a novel magnetic field meter based on a CMR-B scalar sensor that eliminates parasitic EMF and can measure magnetic field pulses with a duration of microseconds. Two hardware solutions for this meter are proposed, as well as the design of a sensor capable of measuring high amplitude magnetic field pulses in very compact volumes. In addition, a study of nanostructured lanthanum-manganite La_1−x_Sr_x_Mn_y_O_3_ films grown with different Mn excess (y) is presented to optimize their parameters for use in magnetic field sensors.

## 2. Design of Microsecond High-Amplitude Magnetic Field Meter

The developed microsecond high-amplitude magnetic field meter includes a magnetic field probe with a magnetic field sensor, a bipolar pulse voltage generator for sensor supply, the amplifier of the sensor’s signal, an analog-to-digital converter, a microcontroller, a USB and a fiber optic interface for communication with the personal computer. A more detailed explanation of each separate unit will be provided in the following sections.

To properly compensate for the impact of the EMF on the sensor’s signal, a high-frequency bipolar voltage of several MHz has to be used for the sensor’s supply. One of the problems that arises when using high-frequency pulses to power a sensor is the distortion of the pulse shape in the line through which the pulses are transmitted to the sensor. Two versions of the microsecond high-amplitude magnetic field meter were developed in this work. In the first version, the amplifier of the sensor’s signal was placed in a separate unit, and the distance between the amplifier and the sensor was 15 cm. Using this version, it was possible to perform magnetic field measurements correctly when the distance between the sensor and the measurement unit of the meter was more than 1 m. In the second version of the magnetic field meter, the amplifier was placed in the measurement unit of the meter. In this case, the correct measurement was possible when the distance between the sensor and the measurement unit was no more than 0.5 m.

### 2.1. Design of the Magnetic Field Probe Using CMR-B-Scalar Sensor

One of the most important tasks in the development of the magnetic field measuring probe was the selection of a suitable sensor. As already mentioned, a sensor based on manganite films, exhibiting the colossal magnetoresistance (CMR) effect, was chosen for this purpose. The electrical and magnetic properties of these films are largely determined by their chemical composition. Previous studies have shown that increasing the Mn content in La_1−x_Sr_x_Mn_y_O_3_ films decreases the electrical resistivity and shifts the temperature of maximum resistance higher [29,30]. In addition, the Sr concentration in these films significantly affects both the resistance and the temperature dependence of their properties [29].

In this work, a series of La_1−x_Sr_x_Mn_y_O_3_ films with a thickness of 350 nm were deposited on a polycrystalline Al_2_O_3_ substrate using the pulsed-injection metal-organic chemical vapor deposition (PI-MOCVD) technique. A detailed description of the film growth techniques and processes can be found in [31]. Taking into account that for polycrystalline films the maximum insulator-metal transition temperature could be achieved with Sr content *x* ≈ 0.2 [30] and Mn excess *y* ≈ (1.10–1.15), we chose to study the main characteristics of the composition La_0.8_Sr_0.2_Mn_y_O_3_ with a different Mn excess.

The temperature dependence of the resistivity (*ρ*) was investigated using a closed-loop helium gas cryocooler over the temperature range of 77–310 K. The magnetoresistance (*MR*) was defined as *MR* = [*ρ*(*B*)/*ρ*(0) − 1] × 100%, where *ρ*(*B*) and *ρ*(0) represent the resistivity in the presence and absence of a magnetic field, respectively. *MR* measurements were performed in the temperature range of 80–350 K using magnetic field pulses with a duration of 1 ms and amplitudes of up to 20 T generated by a non-destructive pulsed magnet powered by a capacitor bank discharging through a multishot magnetic field coil [32].

**Figure 1:**
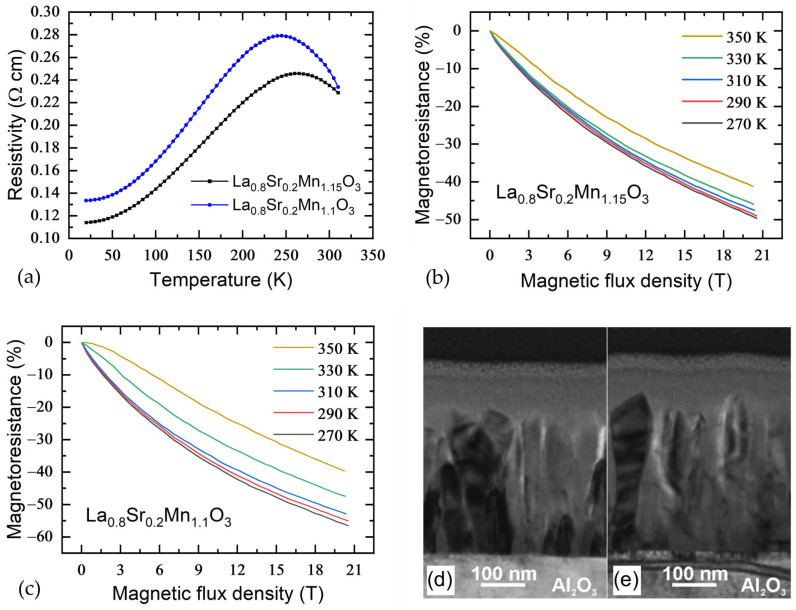
(**a**) Resistivity vs. temperature dependences of LSMO films with different Mn contents. *MR* dependences on magnetic flux density for films with Mn excess *y* = 1.15 (**b**) and *y* = 1.10 (**c**) contents at various ambient temperatures. Cross-sectional bright-field TEM image of the film with *y* = 1.10 (**d**) and *y* = 1.15 (**e**).

The dependences of resistivity on temperature for La_0.8_Sr_0.2_Mn_1.1_O_3_ and La_0.8_Sr_0.2_Mn_1.15_O_3_ films are shown in Figure 1a. One can see that films with a Mn content of *y* = 1.15 have a maximum resistivity of *ρ*_m_= 0.245 Ωcm at the insulator–metal transition temperature *T*_m_ = 270 K. In comparison, the *T*_m_ of films with a Mn content of *y* = 1.1 shifted to a lower temperature of 240 K, accompanied by a higher *ρ*_m_ = 0.28 Ωcm.

The *MR* dependence on magnetic flux density (*B*) for LSMO films with different Mn concentrations at different temperatures is shown in Figure 1b,c. At 270 K, films with *y* = 1.15 show the highest *MR* magnitude compared to those with *y* = 1.1. However, at higher temperatures up to 350 K, the trend is reversed and the films with *y* = 1.15 show slightly higher MR values.

To investigate the cause of the changes in the electrical and magnetic properties of the films, their crystal structure was analyzed using transmission electron microscopy (TEM). Figure 1d,e shows TEM cross-sectional low magnification images of films with different compositions. These images indicate that the films consist of crystallite columns extending across the entire film thickness with their long axes oriented perpendicular to the substrate. However, no significant structural differences, such as variations in column width or grain boundary area, were observed between films with different chemical compositions (Mn excess). In addition, XRD analysis confirmed that there were no structural differences between these films. Consequently, the observed differences in the temperature dependence of the resistivity and magnetoresistance are attributed to changes in the Mn concentration, which lead to variations in the Mn^4+^/Mn^3+^ ratio [33].

Based on the results and considering that the sensors will operate at room temperature and above, films of LSMO with a Mn concentration of 1.15 were selected for the fabrication of magnetic field probes. These films offer a lower resistivity and have a lower temperature dependence of the magnetoresistance.

**Figure 2:**
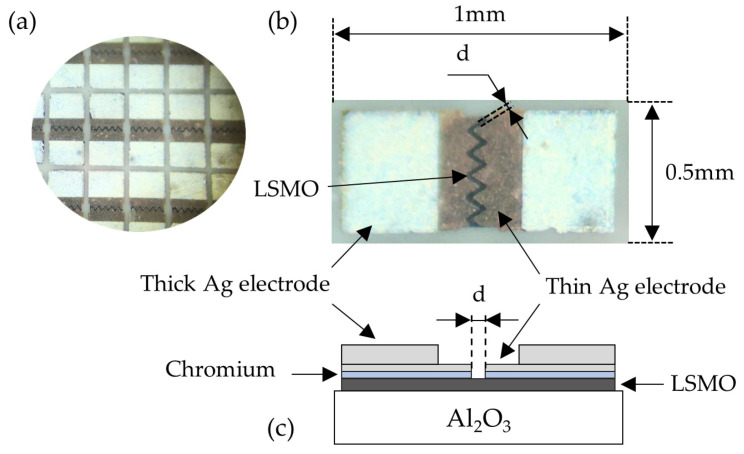
Image of the sensors after photolithography (**a**). Image (**b**) and cross-sectional drawing (**c**) of a single sensor.

For the manufacturing process, the LSMO films deposited on the substrate were formed by using photolithography and chemical etching. Electrodes were then produced by thermally depositing layers of chromium and silver from the gas phase. This process took place in two stages. First, the 20 nm thick Cr layer was deposited to ensure good adhesion between the electrodes and the film, and then a 0.5 μm thick Ag layer was deposited and annealed for 1 h at 450 °C in an Ar atmosphere. The distance *d* between these electrodes was chosen in the range of 5–10 μm (depending on the resistivity of the films) to ensure the resistance of the sample of approximately 500 Ω. For soldering the connecting wires to the sensors, the second silver layer with a thickness of approximately 2 μm was deposited and annealed. Finally, the substrates with the films were cut into sensors with a size of 1 × 0.5 mm^2^. The structure and dimensions of the sensor are shown in Figure 2.

Bifilar twisted wires were then soldered to the sensors. The active area was covered with hot-melt adhesive to protect the film from atmospheric conditions and to reinforce the soldered joints. In addition, the twisted wire cable was shielded against high-frequency noise with a braided sleeve, which was then enclosed in a flexible plastic tube.

**Figure 3:**
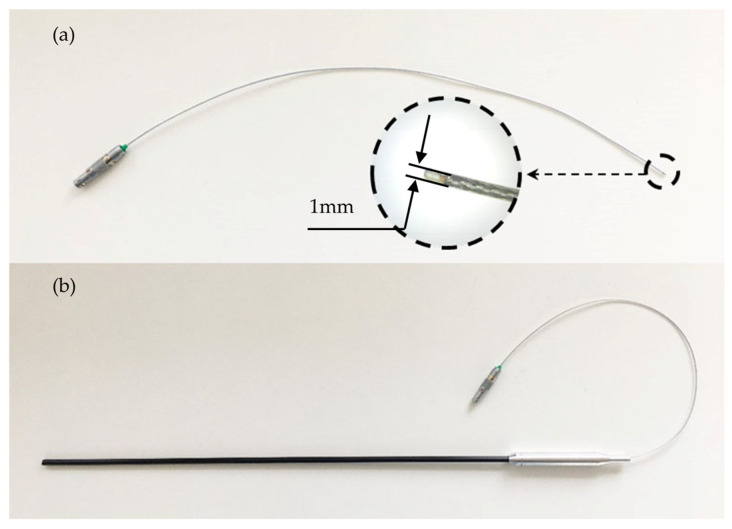
(**a**) Flexible magnetic field probe with length of 25 cm and wire diameter of 1 mm. (**b**) Rigid magnetic field probe in plastic housing with diameter of 3 mm.

Two types of probes were developed: a flexible probe and a rigid probe. The production of a flexible magnetic field probe begins with the preparation of bifilarly twisted copper wires for powering the sensor. Wires with a diameter of 50 µm covered with an insulating material such as varnish, silk or silicone were used for this goal. Next, using solder, a sensor with pre-applied silver contacts was connected to the twisted wires. The soldering process is carried out at a temperature not exceeding 270 °C to avoid overheating of the components. To protect the sensor from environmental influences, it was passivated with thermal adhesives or insulating varnish, thus ensuring durability and longevity. After that, the sensor with wires is placed in a metal screen with a thickness of about 0.1 mm; the screen is designed to suppress external electromagnetic interference. Silicone wire insulation coats the screen and CMR sensor, providing additional protection against high voltage. The probe is connected to the main block of magnetic field meter using a LEMO connector. A view of the flexible magnetic field probe is presented in Figure 3a.

The manufacturing of a magnetic field probe in a rigid housing is the same as that of a flexible probe. The difference is that the rigid probe is housed in a hard plastic tube with a handle. A view of a rigid magnetic field probe is provided in Figure 3b.

In the developed device, the choice of probe type depends on the experimental conditions, with the rigid probe being suitable for situations where it can be easily inserted, while the flexible probe is preferred in cases where access to the measurement area is restricted or requires flexibility.

As already mentioned, to measure the value of the magnetic field, each sensor should be calibrated over a wide range of temperatures and magnetic fields after production. The calibration was performed using a special designed long-pulse high magnetic field generator, which generates a semi-sinusoidal magnetic pulse with a duration of approximately 1 ms and an amplitude of 25 T [32]. The magnetic field in this case was measured using a precisely calibrated B-dot sensor. The pulse duration was chosen to avoid the induction of electromagnetic force in the sensor, which is proportional to the time derivative of the magnetic field. The calibration procedure is described in more detail in [20]. The result of this calibration (the dependence of the resistance of the sensor on the magnetic field at different temperatures) is stored in the memory of the measuring device. When measuring the magnetic field, the microprocessor uses these tables to convert the voltage change across the sensor into a magnetic field value.

### 2.2. Design of the First Version of the Magnetic Field Meter

**Figure 4:**
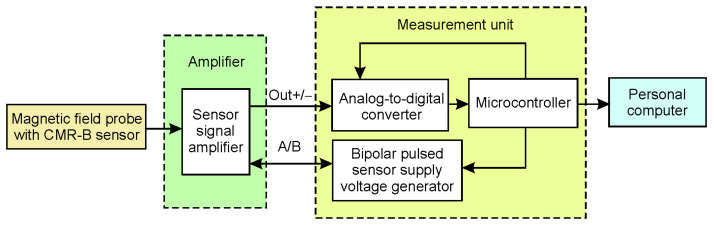
The block diagram of the first version of the magnetic field meter.

Figure 4 presents the block diagram of the first version of the magnetic field meter. In this version, the sensor signal amplifier is a separate unit located near the sensor. In this case, the measurement unit of the magnetic field meter can be several meters away from the sensor. The measurement unit includes a bipolar pulse voltage generator, an analog-to-digital converter, a microcontroller, a USB, and a fiber optic interface for the personal computer.

**Figure 5:**
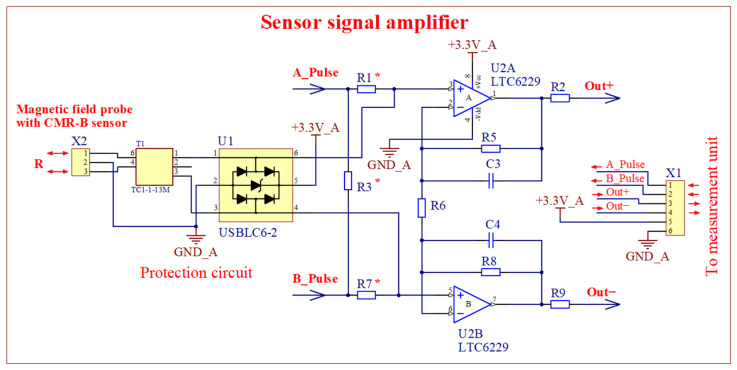
Circuit diagrams of the sensor’s signal amplifier and interference protection circuit of the first version meter. The resistors with the asterisk (∗) are chosen depending on the sensor resistance. The amplifier and protection circuit are located in a separate unit (see Figure 4).

A circuit diagram of the sensor’s signal amplifier is presented in Figure 5. It consists of two parts: the amplifier itself and the interference protection circuit U1 that protects against interference surges. The sensor signal enters the interference protection circuit through a transformer T1, from which it is transmitted to the amplifier. Resistors R1* and R7* are used to adjust the output signal levels of operational amplifiers U2A and U2B for the ADC (maximum input voltage of ADC is 1.875 V, minimum—0.625 V). The value of the voltage across the sensor and its change under the influence of a magnetic field depend on the ratio between the sensor’s resistance, the reference resistors R1* and R7*, and the resistors connected in parallel to the sensor R3*. When selecting the values of these resistors, it is necessary to take into account the maximum allowed input voltage of the operational amplifiers. In addition, it is known that the magnetic field reduces the sensor resistance, but when the measurement temperature decreases, the sensor resistance and voltage increase, which can lead to the saturation of the amplifiers. The main advantage of a magnetic field meter with a sensor signal amplifier that is a separate block close to the sensor is the ability to transmit the amplified signal over a relatively long distance without distortion. The main disadvantage of this solution is that the greater the distance between the main unit and the amplifier, the more complicated the synchronization, which reduces the measurement accuracy.

**Figure 6:**
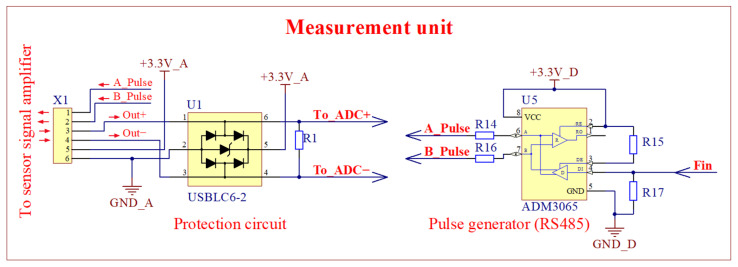
Circuit diagrams of interference protection circuit and bipolar pulsed voltage supply source of the first version of the meter. The bipolar pulse generator and input protection circuit are located in the measurement unit (see Figure 4).

The remaining parts of the meter are placed in another block, including a bipolar pulsed voltage supply, an interference protection circuit, an ADC, a microcontroller, a USB, and fiber optic interfaces for the computer (Figure 4). The circuit diagrams of the interference protection circuit and bipolar pulsed voltage supply source are presented in Figure 6. Interference protection circuit U1 is used to limit interference surges that occur on the communication line between blocks. The analog signal received from the sensor signal amplifier was converted to the digital one by the 16 bit 25 MS/s ADC and sent to the microcontroller (LPC54606J256BD100E, NXP USA Inc., Austin, TX, USA) for further processing according to Equation (1). The bipolar voltage pulses required for the sensor supply are generated by a specialized RS485 (Texas Instruments, Dallas, TX, USA) generator circuit U5. The frequency of the pulsed voltage is controlled by the microcontroller.

**Figure 7:**
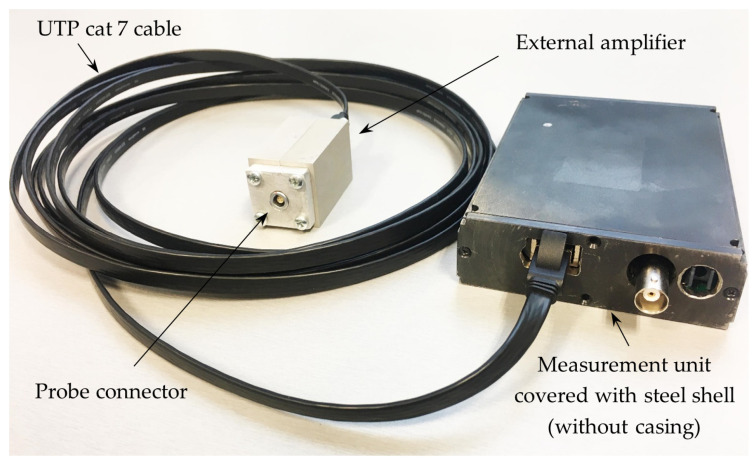
The first version of the magnetic field meter.

A picture of the first version of the magnetic field meter is presented in Figure 7. The meter’s measurement unit is shielded with a 5 mm thick steel shell and connected to a separate amplifier unit via a UTP Cat 7 cable (Belden Wire & Cable B.V., Venlo, The Netherlands). The steel shell is used to suppress external interference that could affect the operation of the measuring device. In the final version, the measurement unit is housed in an aluminum casing. In this version of the meter, the amplifier unit is designed as a separate block that can be positioned close to the sensor (to the measuring location) when the measurement unit is further away. This design offers the advantage that the meter can be used when it is not possible to place the measurement unit close to the measurement object. In addition, studies have shown that this version does not require very precise matching of reference resistors to the sensor resistance. Therefore, the magnetic field sensors with resistance in the range from 500 Ω to 1.5 kΩ can be used in this version of the meter.

### 2.3. Design of the Second Version of the Magnetic Field Meter

**Figure 8:**
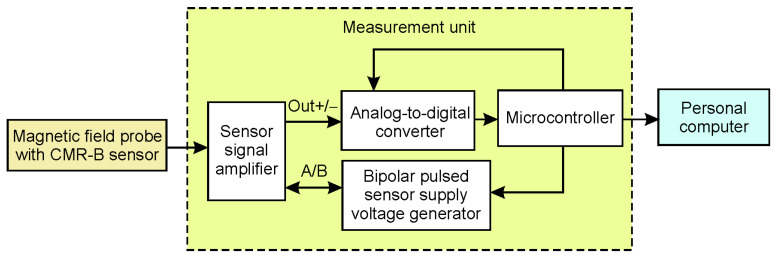
The block diagram of the second version of the pulsed magnetic field meter.

A block diagram of the second version of the pulsed magnetic field meter is shown in Figure 8. It consists of the same components as the first version of the meter. This version differs from the first by integrating the sensor’s signal amplifier in a measurement unit that contains the interference protection circuit ADC, a microcontroller, a bipolar pulsed voltage supply circuit, a USB, and fiber optic interfaces for the computer.

**Figure 9:**
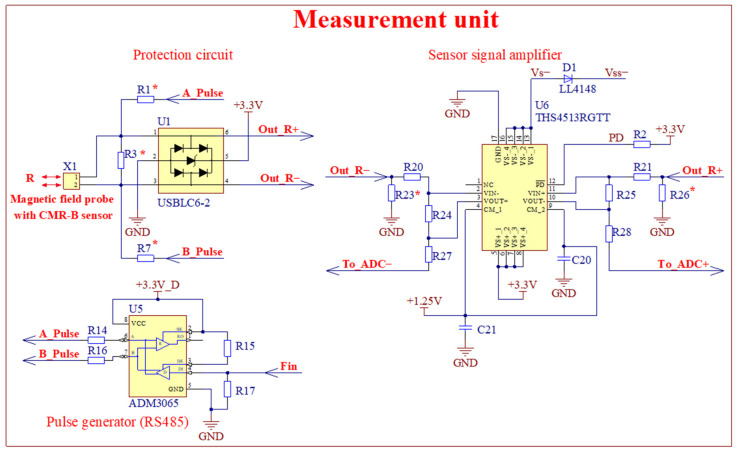
Circuit diagrams of interference protection circuit, bipolar pulsed voltage supply source, and sensor signal amplifier of the second version of the meter. The resistors with the asterisk (∗) are chosen depending on the sensor resistance. The input protection circuit, signal amplifier, and bipolar pulse generator are located in the measurement unit (see Figure 8).

The interference protection circuit, bipolar pulsed voltage supply, and the sensor signal amplifier are mounted in the main unit on a PCB. Their circuit diagrams, which differ from those used in the first version, are shown in Figure 9.

The magnetic field sensor’s signal is fed to the integrated circuit U1 (USLBC6-2, STMicroelectronics, Geneva, Switzerland), which is designed to protect the sensor signal amplifiers from overvoltage (Figure 9). The output signal from U1 (Out R+ and Out R−) is transmitted to the sensor signal amplifier U6 (operational amplifier THS4513RGTT, Texas Instruments, Dallas, TX, USA). This amplifier is characterized by a very low noise level of 2.2 nV/Hz and a fast response time of 2.9 ns. This allows for the high suppression of common interference that affects the magnetic field sensor and the cable connecting it to the magnetic field meter. In addition, this circuit allows the signal to be amplified with extremely low intermodulation distortion, suppressing common-mode parasitic signals up to 90 dB. The output signals from U6 (To_ADC+ and To_ADC−) are transmitted to a 16-bit ADC. As in the first circuit, the magnetic field sensor is powered by a pulsed bipolar voltage generated by the circuit U5 (ADM3065, Analog Devices Inc. Wilmington, MA, USA). As we can see in this circuit, a resistor R3* is connected in parallel to the sensor, the function of which is to reduce the magnetic field probe impedance. This allows us to shorten the duration of the pulse edges, but at the same time reduce the sensitivity of the sensor. This sensitivity reduction can be compensated by changing the sensor signal amplifier’s gain.

To effectively use the advantages of applying a bipolar voltage power supply for the sensor, the sensor supply voltage period has to be significantly shorter than the duration of the magnetic pulse. The ADC with a sampling rate of 25 MS/s was used to measure microsecond duration magnetic field pulses with high time resolution. To obtain a single value of the sensor signal, it is necessary to measure the signal at two points (one when the supply voltage is positive, and the other when it is negative). In this case, the maximum frequency of the pulsed voltage source will be 12.5 MHz. Since the microcontroller buffer can record up to 2.5 kS of values with the pulsed voltage source operating at a frequency of 12.5 MHz, the maximum duration of the recorded magnetic field pulse is 200 μs. Meanwhile, to measure longer pulses, it is necessary to reduce the frequency of the pulsed voltage source and, accordingly, the sampling time of the ADC. 

It must be noted that the shape of voltage pulses supplied to the sensor is distorted. This is related to the compatibility of the transmission line and sensor parameters. One such parameter is the transmission line capacitance. Due to this capacitance, when a rectangular pulse is applied from the power source, the rising and falling voltage edges supplied to the sensor become exponential in shape. This leads to the fact that in an unbalanced supply line, when the duration of the supply voltage pulse is low, the voltage across the sensor may not reach its amplitude value. In this case, the sensor signal will be measured incorrectly. Therefore, to avoid this, it is necessary to use a supply line with the lowest possible capacitance and, accordingly, the sensor’s resistance must be close in magnitude to the impedance of the line. Our measurement system uses a potentiometric circuit; therefore, when measuring the magnetic field, and to obtain the maximum value of the sensor’s signal, it is necessary to match the parameters of the sensor and references resistors R14 and R16. The disadvantage of this second version of the meter is that the meter only operates properly with sensors with less than 650 Ω resistance.

For both versions of the magnetic field meter, after the ADC converts the analog negative and positive peak-to-peak voltage across the sensor to digital form, the microcontroller processes these data using Equation (1) and records the results. This processing eliminates the EMF from the measured signal, leaving only the voltage change caused by variations in the magnetic field. The processed voltage change is then converted into the corresponding magnetic field value and stored.

It should be noted that the sensor’s response is not a linear function of the magnetic flux density (*B*). It also depends on the ambient temperature. It is, therefore, necessary to calibrate the sensors to assign a value of *B* for the resistance change induced by the magnetic field Δ*R* (voltage across the sensor). The calibration tables for the manufactured sensors are stored in the same measuring module.

**Figure 10:**
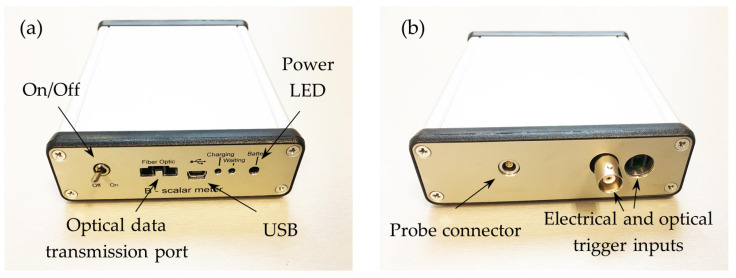
Second version of the measurement unit of the magnetic field meter: (**a**) front side; (**b**) rear side.

**Figure 11:**
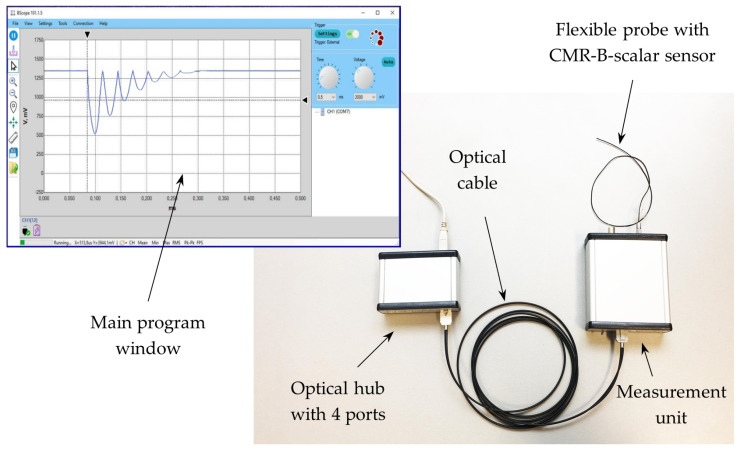
Magnetic field meter and picture of main window of a personal computer interface.

The picture of the second version of the measurement unit of magnetic field meter is shown in Figure 10. It is shielded with a steel shell and covered with an aluminum casing. The power switch, optic fiber connection port, USB port, and battery charge indicator are located on the front panel of the meter. The rear panel of the meter contains a LEMO connector for plug-in of the probe and synchronization connectors (optical and electrical) for receiving an external signal from a trigger. The trigger signal determines the start time of data recording into the meter buffer. A four-pin LEMO connector provides a reliable and fast probe connection, ensuring high signal quality, environmental resistance, and durability, making it ideal for use in complex measurement systems. A view of the magnetic field meter with the probe and a main window of the personal computer interface are presented in Figure 11.

## 3. Results and Discussion

### 3.1. Bipolar Pulsed Voltage Behavior in Different Magnetic Field Meters

**Figure 12:**
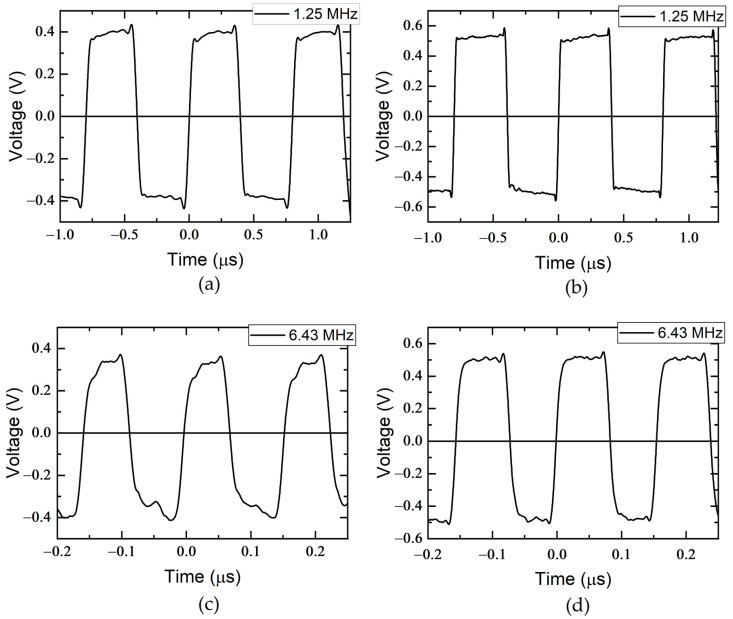
Transients of bipolar pulsed supply voltage across the sensor for the first (**a**,**c**) and second (**b**,**d**) versions of magnetic field meter at various pulsed voltage frequencies.

The shape of the bipolar pulsed supply voltage across the sensor was investigated for both versions of the magnetic field meter. The investigation was conducted at pulsed voltage frequencies of 1.25 MHz and 6.43 MHz. The resulting pulses are shown in Figure 12. It can be observed that the first version of the magnetic field meter has a stronger distortion at the leading and falling edges of the voltage waveform across the sensor, and the rise and fall times of the pulse are 20 ns and 18 ns, respectively. This can be attributed to the higher impedance of the longer cable, including the increased resistance, inductance, and capacitance distorting the signal. In addition, the longer cable is more susceptible to electromagnetic interference (EMI) and can cause signal reflections due to impedance mismatch. These factors combine to distort the quality of the bipolar square wave voltage, while the shorter cable minimizes these effects and preserves signal integrity. The advantage of the first measuring system, however, is the possibility of placing the measuring module far away from the source of the magnetic field. In the second version, the rise and fall times of the voltage pulses are shorter and correspond to 14 ns and 16 ns, respectively.

### 3.2. Testing of the Magnetic Field Meters

**Figure 13:**
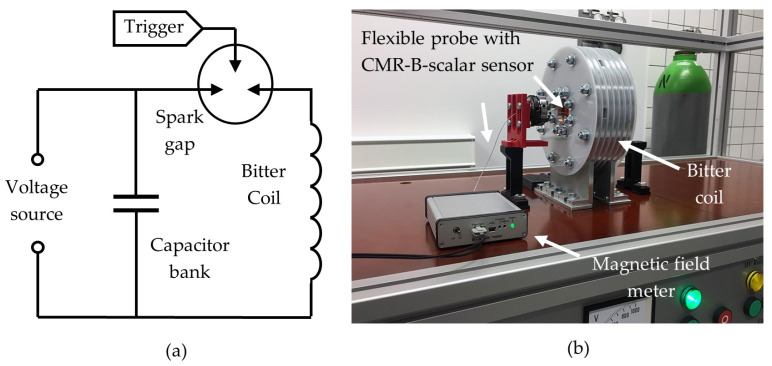
(**a**) Circuit diagram of microsecond magnetic pulse generator which consists of a capacitor bank, a Bitter coil, and a spark gap. (**b**) General view of the experimental setup for testing the magnetic field meter (second version).

**Figure 14:**
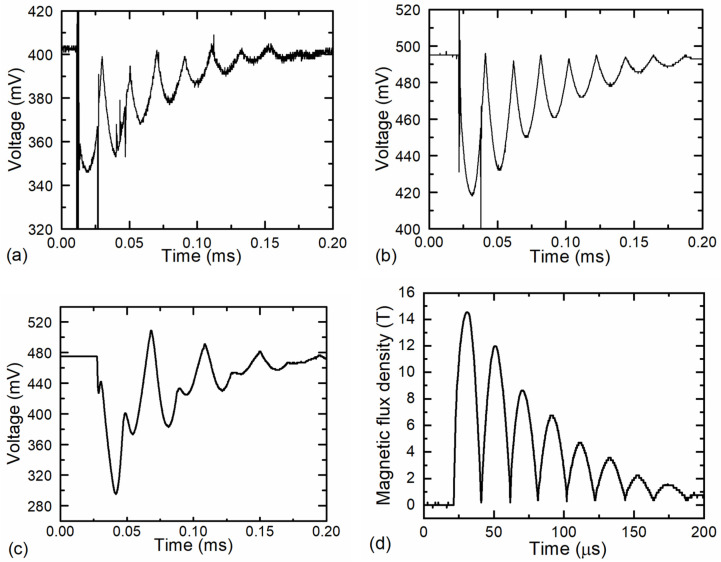
Transients of sensor signal using first (**a**) and second (**b**) versions of magnetic field meter, when sensors are placed in a Bitter coil and magnetic pulse generator capacitors are discharged through it when the capacitors’ voltage is 12.5 kV. (**c**) Transients of sensor signal are detected when useful signal, and EMF is also detected. (**d**) Magnetic flux density in the Bitter coil as a function of time, measured with the second version of the magnetic field meter when the capacitors were charged to 12.5 kV.

A microsecond magnetic pulse generator, which generated pulses that simulate the pulsed magnetic welding equipment pulses, was designed to test the developed magnetic field meters. The circuit diagram of the generator is shown in Figure 13a. It consists of a high-voltage power supply (up to 20 kV), a 40 μF capacitor bank, a spark gap, and a 250 nH Bitter coil with five windings. The generator produces a sinusoidal magnetic field with a decaying amplitude, when the half-period of the magnetic field was about 20 μs. During the test, the sensors were positioned at the center of the Bitter coil, as shown in Figure 13b. After the capacitors were charged, they were discharged via the spark gap through the Bitter coil. The magnetic field generated in the coil changed the resistance of the sensor, which led to a voltage drop that was recorded and processed by the measuring system. Figure 14a,b illustrates the voltage change after the microprocessor has processed the measured signal for these two measurement systems. For comparison, Figure 14c shows the voltage across the sensor when it is supplied with a constant voltage (measurements were made using other devices).

It must be noted that when the sensor is supplied with a constant voltage, the signal at the output of the probe consists of a voltage change caused by the change in the resistance of the sensor under the application of a magnetic field, as well as a voltage induced by the electromotive force in the probe leads. On the other hand, when the sensors are supplied with a bipolar voltage, the contribution of the electromotive force is eliminated by the signal processing, so that only the voltage change caused by the magnetic field remains.

The measurement results show that the signals obtained with the first and second versions of the measuring device are slightly different. Both versions of the meter recognized the triggering when the spark gap trigger was activated; however, the first version of the magnetic field meter is more sensitive to electromagnetic interference. In particular, the high-frequency noise signal generated by the spark gap during switching or reclosing disturbs and distorts the primary signal. These noise signals are visible during the entire measured pulse. In contrast, the signal measured with the second prototype is much cleaner and contains only minimal noise. This difference is because in the first version, the signal is transmitted from the amplifier unit to the main unit via a long cable that is more susceptible to interference. Despite these differences, both measuring systems are capable of accurately measuring short magnetic pulses. In addition, it should be noted that the proposed method of eliminating parasitic signals induced in the sensor wires is effective only when the frequency of these signals is lower than the frequency of the sensor supply voltage or when their duration is several times longer than the length of the power supply pulse. In our case, the voltage at the sensor is distorted by a high-frequency electromagnetic signal of short duration generated by the spark gap, which cannot be eliminated by supplying the sensor with a bipolar voltage of the lower frequency. The only way to attenuate this distortion is to improve the shielding of the probe cable.

An example of a measurement of the magnetic flux density in the Bitter coil is shown in Figure 14d. In this case, the second version of the magnetic field meter was used. The observed change in magnetic inductance results from the discharge of a capacitor bank charged up to 12.5 kV through the Bitter coil. During the measurement, the measurement system recorded both positive and negative voltages across the sensor. The microprocessor then processed this voltage according to Equation (1) and converted it into a resistance. Then, this resistance was converted into the magnetic field by selecting the appropriate calibration curve from the calibration table. The resistance of the sensor measured before applying the magnetic field was used to determine its temperature. As mentioned previously, the CMR-B-scalar sensor measures only the absolute value of the magnetic flux density *B*, and thus the obtained waveform of the magnetic field is unipolar. It can be seen that the maximum magnetic field in the Bitter coil reaches approximately 15 T when the capacitor bank is charged to 12.5 kV.

## 4. Conclusions

A system for accurately measuring short pulsed high amplitude magnetic fields was developed, focusing on eliminating electromotive forces (EMF) by supplying the sensor with a bipolar square wave voltage. By processing the positive and negative voltages measured across the sensor, the EMFs induced in the wires connecting the sensor to the measurement unit were effectively eliminated. This approach successfully mitigates the effects of interfering signals and ensures that the measurements only reflect the changes caused by the magnetic field.

Two versions of the magnetic field meter have been developed and tested, each offering specific advantages under different measurement conditions. The first version, which includes an external signal amplification unit, is suitable for applications that require a large distance between the magnetic field sensor and the measuring device. The second version, where the amplification unit is integrated into the main measuring unit, is ideal for situations in which the measuring unit can be positioned close to the sensor. The probe length of the second version is limited to a maximum of 0.5 m; however, it provides more accurate output signals and better noise suppression.

A specially designed magnetic field sensor based on the Colossal Magnetoresistance effect was developed for both meter versions. The design of the magnetic field probe, which uses nanostructured La_1−x_Sr_x_Mn_y_O_3_ films, was crucial to the meter’s success. By selecting films with a higher manganese content (*y* = 1.15), the resistivity of the sensor was reduced, ensuring compatibility with the impedance of the probe’s transmission cable. The manufacturing process, which used photolithography and chemical etching, enabled high precision in the production of the sensors. In addition, two types of probes—one rigid and one flexible—were developed, which can be adapted to different experimental setups and enable versatile measurement applications.

The final design of the magnetic field meter, which combines advanced sensor technology, signal processing, and customizable probe options, provides a robust solution for the precise measurement of high-amplitude, microsecond-duration magnetic fields. Both versions of the magnetic field meter have been successfully tested by measuring short, high-amplitude magnetic pulses, with the second version demonstrating superior performance in high-interference environments. The system’s ability to operate in magnetic fields up to 20 T, together with its high temporal resolution, makes it applicable to various scientific and industrial fields, including diagnostics during pulsed magnetic field welding and pulsed magnetic field research.

## Data Availability

Data is contained within the article.
